# The Nucleus Accumbens: A Common Target in the Comorbidity of Depression and Addiction

**DOI:** 10.3389/fncir.2020.00037

**Published:** 2020-06-30

**Authors:** Le Xu, Jun Nan, Yan Lan

**Affiliations:** ^1^Department of Physiology and Pathophysiology, College of Medicine, Yanbian University School of Medicine, Yanji City, China; ^2^Department of Orthopedics, Affiliated Hospital of Yanbian University, Yanji City, China

**Keywords:** nucleus accumbens, depression, addiction, comorbidity, deep brain stimulation, dopamine

## Abstract

The comorbidity of depression and addiction has become a serious public health issue, and the relationship between these two disorders and their potential mechanisms has attracted extensive attention. Numerous studies have suggested that depression and addiction share common mechanisms and anatomical pathways. The nucleus accumbens (NAc) has long been considered a key brain region for regulating many behaviors, especially those related to depression and addiction. In this review article, we focus on the association between addiction and depression, highlighting the potential mediating role of the NAc in this comorbidity *via* the regulation of changes in the neural circuits and molecular signaling. To clarify the mechanisms underlying this association, we summarize evidence from overlapping reward neurocircuitry, the resemblance of cellular and molecular mechanisms, and common treatments. Understanding the interplay between these disorders should help guide clinical comorbidity prevention and the search for a new target for comorbidity treatment.

## Introduction

According to epidemiological investigations, 32% of individuals with substance use disorder in America have a comorbid major depressive disorder (MDD; Carey, 2019). People with mental disorders are almost four times more likely to die from a drug overdose, as reported in a meta-analysis of risk factors for prescription overdose (Brady et al., 2017). Depression increases the risk of relapse after withdrawal (Goesling et al., 2015; Sullivan, 2016; Feingold et al., 2018), and, simultaneously, the severity and duration of depressive symptoms in MDD are greater if people who suffer from MDD also suffer addiction (Scherrer et al., 2016a,b). The effect of depression on addiction may be related to the type of drug used, dose of the drug, duration of drug use, socioeconomic position, and psychopathological impairment (Erfan et al., 2010; Merrill et al., 2012; Fink et al., 2015; Scherrer et al., 2016a; Anand et al., 2019; Peters and Soyka, 2019). Recent studies have suggested that etiology of this comorbidity, although poorly understood, reflects an imbalanced activation of the reward circuit and signaling mechanisms (Fink et al., 2015).

From a neurotoxicology point of view, drug abuse and depression can be interpreted as disorders that impair reward-related learning (Volman et al., 2013). The reward-related learning system is strongly conserved in mammals and, given that it enhances survival behaviors such as reproduction and feeding, it represents an important evolutionary advantage (Hyman et al., 2006). One of the most typical symptoms of depression is anhedonia, defined as the loss of pleasure resulting in a general lack of motivation, which could lead to the death of the individual (Der-Avakian and Markou, 2012). On the contrary, the addictive behavior is the opposite because the individual is highly motivated by the object they are addicted to (Hyman et al., 2006).

Multiple studies have shown that the nucleus accumbens (NAc) is strongly associated with a variety of disorders, especially addiction and depression (Qi et al., 2011; Gao et al., 2012; Larson et al., 2015). According to Arango-Lievano et al. (2014) study, the NAc regulates emotional and reward related-stimuli by further integrating signals from different regions of the limbic system. Several animal models of drug dependence and depression have chosen the NAc as the main subject of research (Gao et al., 2012; Amchova et al., 2014; Crofton et al., 2017). Although invasive, deep brain stimulation (DBS) therapy of the NAc has been reported to be effective in treating addiction (Pierce and Vassoler, 2013) and depression (Bewernick et al., 2010). Given that these two disorders can be treated by common NAc-targeting therapies, this may indicate that they have the same or similar pathogenesis. Therefore, the NAc remains a central and critical area to elucidate the common or similar underlying molecular mechanisms of addiction and depression (Arango-Lievano and Kaplitt, 2015). In this review article, we will discuss the neuroanatomical profile and the neural circuits of the NAc as a potential explanation for the comorbidity of addiction and depression. Second, we will elaborate on the various potential mechanisms, such as the function of transmitters, synaptic plasticity, and intracellular signal transduction, and will explain the important role of the NAc in the pathogenesis of comorbidity. Finally, we will discuss the drug and surgical treatment methods for this comorbidity. In summary, this review article aimed to identify the causes and effects of the comorbidity of depression and addiction by investigating the NAc, a key brain region, so that more effective treatment strategies can be developed.

## The Role of the NAc in the Comorbidity of Depression and Addiction

### General Profile of the NAc

As a significant component of the ventral striatum, the NAc is divided into the shell and the core areas. These two areas have different connections and functions. The shell receives information from the limbic system, while the core mainly receives information from the motor system (Park et al., 2019). According to morphological studies, the NAc is different in humans and rats (Salgado and Kaplitt, 2015). In humans, the core region displays a lower density of neurons than does the shell. While it contains some multipolar neurons, it is mainly composed of pyramidal-like neurons with spines on secondary branches. The shell, on the other hand, contains primarily well-arborized multipolar and fusiform neurons (Sazdanovic et al., 2011). In contrast, the shell in rats contains smaller cells with fewer dendrites and dendritic spines than those found in the core (Meredith et al., 1992). In the NAc, medium spiny neurons (MSNs) make up 90–95% of all neurons (Meredith, 1999; Castro and Bruchas, 2019). The remainder is the local circuit interneurons (Kawaguchi et al., 1995). These contain fast-spiking interneurons (FSIs), persistent low threshold and somatostatin-releasing interneurons (SOMs), and cholinergic interneurons (ChIs), the so-called tonically active neurons (TANs; Kawaguchi, 1993; Berke, 2011; Tepper et al., 2018; Castro and Bruchas, 2019). MSNs and TANs are two major groups of neurons in the NAc that are important for studying the comorbidity of addiction and depression (Crofton et al., 2017). MSNs, which are divided into two types according to dopamine (DA) receptor expression, are gamma-Aminobutyric acid (GABA)ergic projection neurons (Cooper et al., 2017). Generally, DA receptor D1 positive (D1^+^) MSNs stimulate reward-related behavior while DA receptor D2 positive (D2^+^) MSNs promote aversive behavior (Turner et al., 2018b). Although, TANs account for only a small percentage (1–2%) of the population of neurons in the NAc, they can regulate MSN activity (Crofton et al., 2017; Collins et al., 2019). Due to its unique location and structure, there is no doubt that the NAc plays a critical role in a variety of motivational and emotional disorders, including depression, drug abuse, and addiction (Salgado and Kaplitt, 2015).

### NAc Circuitry in the Comorbidity of Depression and Addiction

**Figure 1:**
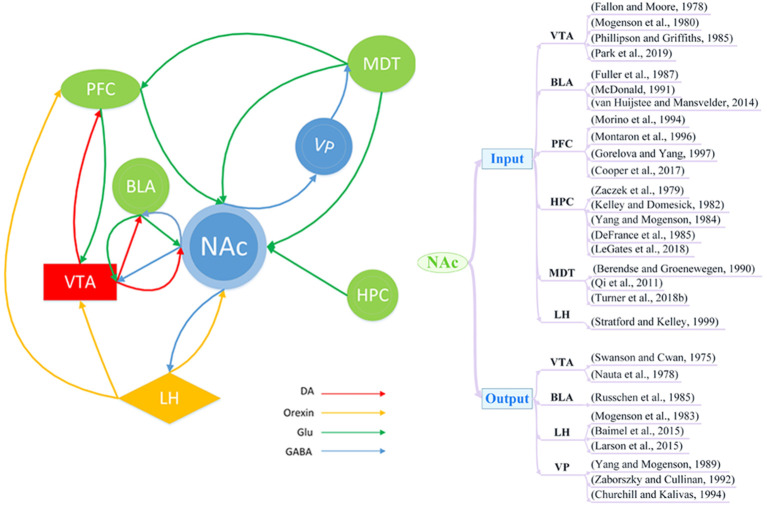
Schematic of brain circuitry implicated in the comorbidity of depression and addiction. Dopaminergic (DA; red), glutamatergic (Glu; green), and orexin neuron (yellow) inputs converge on γ-aminobutyric acid (GABA)ergic (blue) neurons in the nucleus accumbens (NAc) to coordinate and regulate behaviors of depression and addiction. BLA, basolateral amygdala; LH, lateral hypothalamus; MDT, mediodorsal thalamus; PFC, prefrontal cortex; HPC, hippocampus; VP, ventral pallidum; VTA, ventral tegmental area.

The NAc receives complex inputs and outputs from multiple brain regions that are closely related to addiction and depression. It primarily receives inputs in the form of mesolimbic dopaminergic projections from the ventral tegmental area (VTA; Fallon and Moore, 1978; Mogenson et al., 1980; Phillipson and Griffiths, 1985; Park et al., 2019) In addition to DA input, the NAc also receives input *via* glutamatergic projections from the basolateral amygdala (BLA; Fuller et al., 1987; McDonald, 1991; van Huijstee and Mansvelder, 2014), prefrontal cortex (PFC; Morino et al., 1994; Montaron et al., 1996; Gorelova and Yang, 1997; Cooper et al., 2017), hippocampus (HPC; Zaczek et al., 1979; Kelley and Domesick, 1982; Yang and Mogenson, 1984; DeFrance et al., 1985; Legates et al., 2018), and mediodorsal thalamus (MDT; Berendse and Groenewegen, 1990; Qi et al., 2011; Turner et al., 2018b). There is also evidence to suggest that the NAc may receive orexin projections from the lateral hypothalamus (LH; Stratford and Kelley, 1999). The main output neurons from the NAc are MSNs, which project *via* GABAergic projections to the VTA (Swanson and Cwan, 1975; Nauta et al., 1978), LH (Mogenson et al., 1983; Baimel et al., 2015; Larson et al., 2015), ventral pallidum (VP; Yang and Mogenson, 1989; Zaborszky and Cullinan, 1992; Churchill and Kalivas, 1994), and BLA (Russchen et al., 1985; Figure 1).

Studies of addiction and depression have revealed the involvement of several pathways, such as the VTA-NAc (Hyman et al., 2006; Qi et al., 2011; Lammel et al., 2014; Martinez-Rivera et al., 2017; Boekhoudt et al., 2018; Li et al., 2019), PFC-NAc (Qi et al., 2011; Turner et al., 2018b), HPC-NAc (Legates et al., 2018), and NAc-LH (Larson et al., 2015) pathways. Furthermore, the NAc is also involved in some complex pathways, such as the cortico-striato-pallido-thalamocortical loop (Qi et al., 2011; Crofton et al., 2017). In this circuitry, dopaminergic, glutaminergic, and GABAergic pathways work together to regulate the function of neural circuits. For example, the PFC sends glutamatergic projections to the NAc, and the NAc sends GABAergic projections to the globus pallidum, from which GABAergic projections can project into the MDT. Finally, the MDT sends nerve fibers back to the PFC to complete the loop. Thus, it appears highly likely that depression and addiction have similar pathways of nerve fiber transmission, which could represent a structural basis for their comorbidity.

## Possible Influencing Factors That Mediate the Comorbidity of Addiction and Depression

### The Dopamine System

The mesolimbic DA system in the NAc is known to tune attributes of reward-related behavior by encoding the predictability of a reward, increasing the associative learning of a rewarding context, and creating an incentive, which is salient for reward (Schultz, 1998). The DA system in the NAc is also involved in emotion-related behaviors, particularly behaviors related to depression (Luscher and Malenka, 2011; Lammel et al., 2014; Li et al., 2019). Dopaminergic dysfunction is a common mechanism in a variety of reward and motivational disorders, including addiction and depression (Dubol et al., 2018).

It has been shown that the infusion of a D1 receptor agonist into the NAc can reverse the impaired acquisition of morphine-induced conditioned place preference (CPP) in the chronic mild restraint rat model of depression (Gao et al., 2012). However, the injection of DA receptor antagonists into the NAc, which blocks the VTA DA neuron activation, has been found to cause depression-associated symptoms (Lammel et al., 2014). These results suggest that the transmission of DA in the NAc may be of great importance to the occurrence and treatment of the comorbidity of depression and addiction.

In animal studies, behavioral models are used to imitate addiction and depression. In the olfactory bulbectomy (OBX) rat model of depression, the bilateral ablation of the olfactory bulbs induces depression-like symptoms (Holmes et al., 2002; Smaga et al., 2017). Several studies have used the OBX model with intravenous self-administration of drugs as a model of comorbid addiction and depression (Babinska et al., 2016; Jastrzebska et al., 2017). Amchova et al. (2014) reported that the DA levels in the NAc of OBX rats did not increase in response to self-administration of the cannabinoid CB1 receptor agonist WIN 55, 212-2 compared to a control group. The authors suggested that this was due to a decrease in DA transmission in the NAc during the depressive stage, which led to a decreased response to reward. This diminished reward response may have led to a compensatory increase in drug intake and self-administration (Amchova et al., 2014). Furthermore, Kucerova et al. (2012) reported that OBX animals showed higher methamphetamine intake than their sham counterparts. Another experiment revealed that OBX rats self-administered extremely low doses of amphetamines faster with significantly increased stable self-administration of the low-dose amphetamines compared to that in sham rats (Holmes et al., 2002). These studies support the hypothesis that drug intake is increased in animal models of depression. Furthermore, the results from the study by Frankowska et al. (2014) suggested that depressive-like behavior was associated with greater drug cravings and a higher tendency to relapse, as revealed in rats with combined bilateral OBX and self-administration of intravenous cocaine. Babinska et al. (2016) further improved Frankowska et al.’s (2014) and Amchova et al.’s (2014) experiments on the OBX model of depression combined with drug administration. Babinska et al. (2016) chose the highly translational forced abstinence paradigm in which the animal did not have access to the operant self-administering drug and is kept in the home cage to mimic the human situation as the patient usually discontinues the drug-taking in a different, non-drug-related environment. They report that OBX rats displayed a significantly increased reinstatement of methamphetamine seeking behavior indicating higher vulnerability to relapse and trend towards higher drug intake during the maintenance phase. Yet some indicators, such as the mean number of active nose-pokes, methamphetamine infusions, and drug dose, are stable with only a trend towards an increase in the OBX group in the middle of the maintenance phase. This could be related to the total duration of drug exposure (Babinska et al., 2016).

However, different changes in the DA system have been observed in another depression model. In the social defeat stress (SDS) model, increased DA release occurred in the NAc of rats and mice (Tidey and Miczek, 1996; Han et al., 2015; Holly et al., 2015; Hwa et al., 2016). SDS has also been shown to increase the vulnerability to cocaine self-administration as an increased cocaine intake was observed after experiencing SDS in rats (Miczek and Mutschler, 1996). Furthermore, SDS outside the drug-taking context reliably increased the amount of cocaine self-administered by rats that were given 24-h “binge” access (Boyson et al., 2011; Shimamoto et al., 2015). SDS has also been shown to enhance the sensitivity to cocaine CPP, which is a model of addiction and relapse in mice (Ribeiro Do Couto et al., 2009; Rodríguez-Arias et al., 2017).

In another interesting study that focused on female rats, individual differences in the anhedonic-like response of rats to chronic social defeat stress (CSDS) were found. These differences were associated with subsequent cocaine-taking behavior. Based on their saccharin intake while exposed to stress, the female rats were defined as stress-resistant (SR) or stress-sensitive (SS). The NAc DA response to cocaine challenge was significantly lower in SR rats than in the SS and non-stressed control rats. Females showing reduced saccharin preference during CSDS that later self-administered significantly less cocaine (SS rats) did not exhibit an altered DA response to cocaine compared to the controls. Thus, stress-induced alterations within the mesolimbic DA system cannot fully explain the reduced cocaine self-administration observed in these animals (Shimamoto et al., 2015).

Therefore, changes in the DA system in the NAc alone fails to fully explain the phenomenon that depression may lead to addiction susceptibility. Brain-derived neurotrophic factor (BDNF) and its receptor, tyrosine receptor kinase B (TrkB), is expressed in the mesolimbic DA circuit, which projects from the midbrain DA neurons in the VTA to the NAc in the basal forebrain (Koo et al., 2019). Studies support the involvement of BDNF in the VTA-to-NAc circuit (Cleck et al., 2008; Covington et al., 2011; Miczek et al., 2011; Wang et al., 2016; Li et al., 2017). Elevated BDNF in the NAc has been shown to increase the depressive phenotype (Chaudhury et al., 2015) and cocaine addiction in animal models (Graham et al., 2007, 2009; McGinty et al., 2010; Li and Wolf, 2015; Gueye et al., 2019). A previous study on chronic optogenetic phasic stimulation of the VTA-NAc circuit during SDS exacerbated the defeat-induced behavioral symptoms. These aggravated symptoms were also normalized by a BDNF-TrkB blockade in the NAc, which showed that BDNF-TrkB signaling, rather than DA signaling in the VTA-NAc circuit, is crucial for facilitating depression-like outcomes after experiencing CSDS. This established that BDNF-TrkB signaling is a pathological mechanism during periods of chronic stress (Wook Koo et al., 2016). However, BDNF overexpression in the NAc induced by the environment reduces withdrawal symptoms and craving behavior in a rat model of heroin addiction (Li et al., 2017). Furthermore, BDNF over-expression participates in the regulation of the DA system by up-regulating the dopamine receptor D3 (DRD3) and dopamine transporter (DAT; Li et al., 2017). The BDNF-TrkB signaling pathway may further activate transcription factors to mediate the comorbidity of addiction and depression (Arthur et al., 2004; Levine et al., 2005; Nair and Vaidya, 2006; Vialou et al., 2012).

In clinical studies, the results from the research by Liu et al. (2013) revealed that long-term heroin use leads to a long-term decrease in striatal DAT and that the decrease in DAT may underlie heroin users’ depression; there was a significant correlation between DAT availability and the Hamilton Depression Rating Scale (HDRS) scores in heroin users. Though limited in number, human studies in addicted populations, using various drugs, support the concept that DA transmission is reduced in the brains of drug-dependent subjects, and the response to drug challenge is higher in drug-dependent subjects than in controls (Melis et al., 2005). Increased levels of BDNF protein in the NAc are reported in patients with MDD at autopsy, including individuals who were depressed at the time of death despite taking antidepressants, suggesting there is elevated BDNF signaling in treatment-resistant MDD (Krishnan et al., 2007).

In conclusion, both the OBX model and the SDS model are depression models with good face validity and they both increase the vulnerability to addiction (Babinska et al., 2016; Koo et al., 2019). The release of DA in the NAc decreased in the OBX model but increased in the SDS model. Depression models seem to show that both increased and decreased DA can affect the susceptibility to addiction, which makes us wonder whether the BDNF function is truly involved in the induction of addiction by depression. Therefore, future studies will still need to measure the changes in BDNF in the comorbidity models to further clarify whether BDNF has similar changes with addiction and depression models. This can be useful to evaluate the differences between comorbidity models for the selection of a model with better etiological, face, and predictive validity. Studies are still needed to elucidate the role of BDNF and DA in the comorbidity of addiction and depression. Furthermore, these studies should include female rodents to further explore the influence of sex on the occurrence of this comorbidity. Moreover, few preclinical studies have examined the relationship between depression and addiction in the opposing direction, which begs the question, can a model of drug self-administration potentiate a depressive-like phenotype? In the future, clinical studies should measure the DA content in the NAc of subjects with MDD to determine whether the BDNF content is also increased in patients with different drug addictions. Moreover, according to the relevant mechanisms reported in preclinical studies, whether the change in BDNF can be inhibited to treat comorbid depression and addiction has not been clarified.

### Acetylcholine

Although ChIs in the NAc only account for 1–2% of the neuronal population, they play a dominant role in regulating the activity and function of the NAc (Salgado and Kaplitt, 2015; Cheng et al., 2019; Collins et al., 2019). Dysregulation of cholinergic signaling can lead to addiction (Witten et al., 2010; Lee et al., 2016) and depression (Warner-Schmidt et al., 2012; Crofton et al., 2017).

Animal studies have shown that the capacity for cholinergic signaling is dictated by the availability and activity of the presynaptic, high-affinity choline transporter (CHT; Cheng et al., 2019). Dong et al. (2013) showed that in CHT hemizygous mice (CHT^+/–^ mice), where the gene that encodes CHT is eliminated and there is reduced expression of CHT, there is a significant reduction in extracellular DA levels in the NAc, as measured by *in vivo* microdialysis. Moreover, CHT heterozygosity contributes to a blunted increase in DA after the systematic administration of cocaine. The reduction in cocaine-induced DA elevations in CHT^+/–^ mice may be associated with changes in basal DA or DAT levels/activities. Because DA signaling in rodent models is thought to mimic that in depression (Chaudhury et al., 2013; Tye et al., 2013), decreased CHT expression has been suggested to increase the risk of depression (Dong et al., 2013). Furthermore, Cheng et al. (2019) found that inhibiting ChIs in the NAc caused mice to become more susceptible to stress while increasing ChI activity reversed this depressive phenotype in a model of stress-induced depression. However, these changes still do not fully account for whether CHT^+/–^ mice are associated with the co-occurrence of addiction and depression. Future studies should measure CHT^+/–^ mice for behavioral indicators of depression such as the forced swim and tail suspension tests, which can provide direct evidence that decreased CHT expression can induce depressive-like behavior. Further studies are needed to define the mechanism by which drug addiction may induce a reduction in CHT expression to decrease DA signaling, resulting in the development of depression.

Decreased expression and function of the hyperpolarization-activated cyclic nucleotide-gated channel 2 (HCN2) occurs in the ChIs of the NAc shell in mouse models of depression with p11 conditional knockout (cKO) mice and SDS mice. Furthermore, overexpression of HCN2 channels in the ChIs enhances cell activity sufficiently to rescue depressive phenotypes in mice (Cheng et al., 2019). However, cocaine treatment leads to a significant increase in the expression of the HCN2 subunit in its glycosylated and non-glycosylated protein subtypes, as shown by western blot analysis of rat tissue samples from the VTA, NAc, PFC, and HPC (Santos-Vera et al., 2013). This suggests that the observed mesolimbic excitability changes that occur during cocaine addiction might be associated with alterations in the subunit composition of HCN channels (Santos-Vera et al., 2013). However, whether changes in the HCN2 subunits would impact ChIs in addiction was not reported.

A small receptor-binding protein p11 is a critical regulator of ChI activity within the NAc, as measured by the DA response to the mesolimbic DA reward stimulus pathway (Hanada et al., 2018; Liu et al., 2019). p11 is required for reward-mediated NAc ChI activation and the induction of acetylcholine (ACh) release, resulting in the enhancement of DA release (Hanada et al., 2018). Moreover, p11 in ChIs is shown to be a key regulator of depressive-like behavior. Previous studies have also shown that p11 knockout mice show depressive-like phenotypes, and that the depressive-like behaviors can be rescued by overexpression of p11 in NAc ChIs (Alexander et al., 2010; Warner-Schmidt et al., 2012). The expression of p11 not only plays an important role in depressive behavior but also plays a significant role in addiction. Arango-Lievano et al. showed that p11 knockout mice have enhanced cocaine CPP due to the downregulation of p11 expression in the NAc. In wild-type mice, cocaine decreases the expression of p11 in the NAc, while overexpression of p11 in the NAc can reduce CPP (Arango-Lievano et al., 2014). Therefore, a therapeutic strategy to improve the function and expression of p11 and its signaling pathway in NAc ChIs may hold promise for the treatment of addiction and depression comorbidity.

Glycogen synthase kinase 3 beta (GSK3β) is an uncommon protein serine kinase that participates in several cellular signaling pathways and is associated with multiple rewards and motivational disorders (Xu et al., 2011; Shi et al., 2014; Huang et al., 2015; Patel and Woodgett, 2017; Inkster et al., 2018). The activity of GSK3β in the NAc core mediates the initiation and expression of methamphetamine-induced locomotor sensitization, suggesting that GSK3β may be a potential target for the treatment of psychostimulant addiction. Crofton et al. (2017) used knocked down GSK3β expression with a novel adeno-associated virus vector (AAV2). They found that these GSK3β knockdown rats preferred the same sucrose concentration than controls when familiar with the taste and groomed each other less than controls after a brief separation. Further, GSK3β in the NAc shell increased maintenance responding, a trend toward the increased acquisition, and indicated an interaction of dose and group for dose-response. GSK3β knockdown also led to a significant decrease in spontaneous firing rate compared to the ChIs in control animals. The results suggest that the silencing of GSK3β in the NAc increases depression- and addiction-related behaviors, possibly by decreasing the intrinsic excitability of ChIs. However, this study did not rule out the potential contributions of other neuronal subtypes (Crofton et al., 2017).

In conclusion, cholinergic neurons in the NAc appear to be extremely important in the development of addiction and depression, potentially *via* their ability to change CHT function, HCN2 expression, or p11 expression, as well as silencing GSK3β, and thus may play a role in the comorbidity of these conditions.

Clinical studies are very limited in measuring changes of CHT, HCN2, GSK3β, and p11 in patients with comorbidity. A blinded western blot analysis of extracts from NAc tissue revealed a significant reduction in p11 protein in patients with depression compared with controls (Alexander et al., 2010). Studies in human postmortem brain and peripheral cells also identified correlations between alterations in GSK3β and mood disorders (Jope, 2011).

Although clinical studies are limited, we can presume that CHT, HCN2, GSK3β, and p11 may be important targets for the treatment of comorbid addiction and depression in the future owing to the findings from preclinical studies. However, some problems still need to be resolved. A direct relationship between CHT expression and depression still needs to be established. The overexpression of HCN2 has antidepressant effects; however, the effect of HCN2 expression on the treatment of addiction remains to be explored. GSK3β in the NAc increases depression- and addiction-related behaviors. Whether increasing GSK3β can reverse addiction and depression phenotypes still needs to be tested. Compared with the other three targets, p11 has relatively more evidence to suggest its role in addiction and depression. In animal experiments, the upregulation of p11 expression in the NAc can reduce the conditional position preference caused by cocaine and has an antidepressant effect. A significant decrease in p11 has been found in NAc tissue from patients with depression. Future studies may evaluate whether p11 knock-out mice permitted to self-administer cocaine represent a model of depression and addiction comorbidity with good face validity. Therefore, p11 is likely to be a key target for the treatment of this comorbidity in the future.

### Endogenous Opioid Peptides and Opioid Receptors

Numerous studies have shown that endogenous opioid peptides and their three receptors—mu (MORs), delta (DORs), and kappa (KORs)—are central players in the comorbidity of depression and addiction (Lutz and Kieffer, 2013). It is worth mentioning that the dynorphin-KOR system in the NAc plays a key role in this comorbidity (Al-Hasani et al., 2015; Chartoff et al., 2016). In animal models, systemic administration of a KOR agonist (U-69593) dose-dependently increased immobility in the forced swim test (FST) without specific effects on locomotor activity (Mague et al., 2003). Another study found that the KOR agonist salvinorin A increased intracranial self-stimulation (ICSS) thresholds and significantly lowered the breakpoint on the progressive ratio schedule, indicating a decrease in motivation. It also significantly reduced the phasic release of DA without affecting DA reuptake in the NAc core, as assessed by fast-scan cyclic voltammetry (FSCV; Ebner et al., 2010). Furthermore, the KOR agonist U50, 488 inhibited DA release evoked by either medial forebrain bundle (MFB)- or pedunculopontine tegmental nucleus (PPTg)-induced activation of VTA inputs to the shell or core of the mouse NAc (Ehrich et al., 2014). Administration of U50, 488 over a short time frame (15 min) could block cocaine CPP, and only significantly reduced the effect of cocaine on the DA response evoked by PPTg stimulation of the NAc core. Conversely, U50, 488 administration increased the cocaine CPP over a longer period (60 min) and significantly increased the effects of cocaine on the DA response evoked by either MFB or PPTg stimulation of either the NAc shell or core (Ehrich et al., 2014). Thus, drug activation of KOR can lead to depressive- and addictive-like behaviors, as well as affect the release and response of DA in the NAc. Moreover, activating KOR through photostimulation of dynorphin-expressing cells in the NAc shell, Al-Hasani et al. (2015) found that the ventral and dorsal NAc shell drives opposing behaviors. As such, they reported that dynorphinergic neurons in the ventral shell of the NAc drive aversion, whereas dynorphinergic neurons in the dorsal shell of the NAc drive preference and reward-seeking behavior (Al-Hasani et al., 2015). Although the mechanism by which the KOR mediates these opposing behaviors in two distinct regions of the NAc shell remains unknown, this finding suggests that comorbid depression and addiction may be treated by the use of a KOR receptor antagonist targeting the NAc. A previous study showed that the escalation of drug-seeking behavior was suppressed by the long-acting KOR antagonist norbinaltorphimine (nor-BNI) in a 12-h long self-administration model of heroin addiction in rats (Schlosburg et al., 2013). Furthermore, local injection of nor-BNI in the NAc significantly blocked the expression of prolonged morphine withdrawal-induced depression-like behaviors without influencing general locomotor activity in mice (Zan et al., 2015). The study has also found that prodynorphin (Pdyn) mRNA and protein levels in the NAc were elevated 4 weeks after morphine withdrawal in mice with a chronic escalation morphine regimen that significantly induced depressive-like behavior (Zan et al., 2015). The authors of that study also found that Pdyn mRNA and protein levels in the NAc were elevated 4 weeks after morphine withdrawal in mice with a chronic escalation morphine regimen that significantly induced depressive-like behaviors (Zan et al., 2015). They also demonstrated that the knockdown of Pdyn in the NAc inhibited sensitization to repeated administration and that it also significantly reduced depressive-like behavior (immobility) in the FST. Future studies should measure levels of Pdyn peptide to locate the brain regions in which dynorphin release may be reduced following Pdyn knockdown in the NAc (Cohen et al., 2014).

Chronic cocaine exposure increases the levels of dynorphin, which acts on KORs and inhibits DA release in the NAc to induce negative emotional states after withdrawal (Cohen et al., 2014; Ehrich et al., 2014; Chartoff et al., 2016). In animal studies, chronic drug addiction may activate transcription factors such as CREB and ΔFosB in the NAc (Pliakas et al., 2001; Dinieri et al., 2009; Sim-Selley et al., 2011; Muschamp and Carlezon, 2013; Carlezon and Krystal, 2016). These transcription factors can increase the expression of dynorphin, which can act at the KORs. This in turn causes a reduction in the release of DA in the NAc, which leads to depression or the core symptoms of depression.

In clinical research, Pdyn gene expression and dynorphin tissue levels are elevated in individuals with a history of psychostimulant abuse (Hurd and Herkenham, 1993). It has been shown that KOR agonists induce profound dysphoria when administered to humans (Pfeiffer et al., 1986); thus, reducing KOR system activity may be an effective method to treat addiction and depression. Buprenorphine is not only a partial agonist of the mu-opioid receptor but also a strong KOR antagonist with antidepressant activity (Karp et al., 2014). A recent open-label clinical trial showed that the combination of the non-selective KOR antagonist buprenorphine and mu-opioid antagonist significantly reduced depressive symptoms in patients with treatment-resistant depression (Chavkin and Koob, 2016). Selective KOR blockers should be used in future studies, and further clinical trials are needed to fully establish their effect on comorbid depression and addiction.

In general, activation of KOR in the NAc can lead to addiction and depression, which can be treated with a KOR receptor antagonist. Photostimulation has also aided the identification of different areas in the NAc shell that regulate aversion, preference, and reward-seeking behavior. Thus, future experiments should use microinjection of a KOR receptor antagonist into particular NAc regions, such as the ventral and dorsal NAc shell, to identify the effect of comorbidity treatment among the different areas of the NAc. This will be of great significance for the future selection of injection sites for drugs. Although KOR antagonists have a role in the treatment of both addiction and depression, it is still necessary to apply KOR antagonists in a comorbidity model and compare the treatment effect with that in the separate addiction or depression models to further clarify their effect on the comorbid depression and addiction. Clinical studies on the treatment of comorbid addiction and depression with KOR receptor antagonists are still lacking, but KOR antagonists represent very promising drugs to treat this comorbidity according to the results from preclinical studies.

### Glutamatergic System

Reduced glutamate reuptake capacity appears to be linked to many neuropsychiatric disorders, including addiction and depression (McCullumsmith and Sanacora, 2015). Numerous studies have shown that glutamate, an excitatory neurotransmitter in afferent pathways in the NAc, is involved in reward learning and motivation (Yu et al., 2017; Turner et al., 2018b). Depression and addiction are disorders of varying degrees of reward; excessive reward plays a role in addiction, while anhedonia plays a role in depression. However, there have been few experimental studies examining the role of the neurotransmitter glutamate in the NAc in comorbid depression and addiction.

Glutamate receptors, including α-amino-3-hydroxy-5-methyl-4-isoxazole propionic acid receptors (AMPARs), metabotropic glutamate receptors (mGluRs), and N-methyl-D-aspartate (NMDA) receptors, have long been considered to mediate addiction and depression (Turner et al., 2018a). According to animal studies, VTA Cav1.3 channel activation can promote cocaine-mediated and depressive behaviors by enhancing CP-AMPAR transmission in the NAc. VTA Cav1.3 channels are necessary for a GluA1 S831 phosphorylation-dependent increase in CP-AMPARs in the NAc shell. One mechanism suggested to contribute to increases in CP-AMPAR levels at the synapse is S831-GluA1 phosphorylation by calmodulin-dependent protein kinase II alpha (CaMKIIα). However, the direct relationship between CaMKIIα and CP-AMPAR remains to be established. It has been suggested that Cav1.3/CaMKII/ERK2 signaling within the VTA mediates the long-term molecular changes in the NAc *via* a CREB-dependent mechanism (Martinez-Rivera et al., 2017). Moreover, cocaine CPP and depressive-like phenotypes can be reversed by the infusion of CP-AMPAR-specific blockers into the NAc shell, which suggests that the modulation of AMPAR transmission may link addiction with depression (Martinez-Rivera et al., 2017). Furthermore, a computational model revealed that synaptic plasticity of glutamatergic and dopaminergic neurons affects the reward system in the NAc and is associated with comorbid depression and addiction (Qi et al., 2011). A computational model was set up with an ordinary differential equation of biochemical reactions and signal transduction processes that describe the three components, including DA metabolism in the presynapse, signal transduction in the postsynapse, and trafficking of AMPARs, ultimately connecting DA and glutamate signals to synaptic plasticity of MSNs (Qi et al., 2011). Although this model is helpful for our understanding of the comorbidity of addiction and depression, it is still not complete and lacks a role for Ach and other transmitters. Moreover, this article mainly reported the uses of amphetamine as an addictive drug for research. In the future, the model will require the inclusion of additional addictive drugs like cocaine and morphine to use it to fully explain the complex process of comorbid depression and addiction.

The functions of mGluRs, including group-I (mGluR1/5), group-II (mGluR2/3), and group-III (mGluR4-8), are closely related to the occurrence of addiction and the treatment of depressive symptoms. Intra-Nac shell infusion of the mGluR5 antagonist 3—pyridine (MTEP) has been reported to block cocaine-primed reinstatement and the corresponding cocaine-seeking behavior (Benneyworth et al., 2019). Li et al. (2018) performed *in vivo* microdialysis in rats following cocaine self-administration and extinction training. They found that systemic treatment or local infusion of a mGluR5 antagonist into the Nac reduced cocaine-taking and cocaine-seeking behaviors and the corresponding cocaine-induced glutamate release, but not cocaine-induced DA release (Li et al., 2018). However, mGluR5^−/−^ mice displayed more depressive-like behaviors than control mice following exposure to various stressful stimuli. Notably, targeted pharmacological activation of mGluR5 in the NAc increased ΔFosB expression, which can promote stress resilience and is related to depression (Shin et al., 2015). The experimental results from the study by Qian et al. (2019) indicate that withdrawal-induced mGluR2/3 downregulation alters neural plasticity after morphine exposure in rats, and this mechanism may contribute to drug addiction. Also, several mGluR2/3 antagonists, which block the mGluR2/3 receptor in the NAc, may prevent the depressive symptoms induced by the withdrawal of methamphetamine (Iijima et al., 2013; Jaso et al., 2017). mGluR7 has received much attention as a potential target for the treatment of comorbid depression and addiction (Li et al., 2009). AMN082, a selective mGluR7 antagonist, reduces the development and expression of cocaine and morphine sensitization (Jenda et al., 2015). It attenuates adenylate cyclase/protein kinase A activation, which subsequently attenuates the entry of Ca^2+^ through voltage-dependent Ca^2+^ channels and decreases evoked glutamate release; the results indicate that AMN082 and fluoxetine inhibit glutamate release *via* a common intracellular mechanism (Wang et al., 2018). Thus, the mGluR7 antagonist AMN082 might have therapeutic implications not only in the treatment of cocaine and opioid addiction but also in the treatment of depression.

NMDA receptors are implicated in experience-dependent synaptic changes. Several studies have demonstrated that NMDA receptor activation correlates with drug-induced synaptic changes (Turner et al., 2018a). Accumulating evidence suggests that there is impairment of NMDA receptor-dependent long-term potentiation (LTP) and long-term depression (LTD) at glutamatergic synapses in the NAc of animal models of addiction (Moussawi et al., 2009; Kasanetz et al., 2010; Curcio et al., 2013). However, studies examining NMDA receptor-dependent LTP and LTD at glutamatergic synapses in the NAc of animal models of depression are still lacking. NMDA receptor antagonists have antidepressant and anti-addiction effects in animal models. A single systemic injection of the NMDA receptor antagonist ketamine at an intraperitoneal dose of 10 mg/kg produces a significant reduction in FST immobility shortly after administration (30 min to 1 h), which has been shown to persist for an average of 7 days in both rats and mice (Aleksandrova et al., 2017). What is more, the NMDA receptor antagonist DL-2-Amino-5-phosphonopentanoic acid sodium salt (AP5) can reduce the CPP induced by morphine (Siahposht-Khachaki et al., 2016).

The above preclinical studies suggest that glutamate receptors are implicated addiction and depressive behaviors, which provides a theoretical basis for the future study of glutamate receptor antagonists in the treatment of comorbid depression and addiction. It is also hoped that future studies will focus more on the role of these receptor antagonists in comorbidity models and on the changes in the expression of these receptors in the NAc.

In clinical research, since the rapid and robust antidepressant effects of the NMDA receptor antagonist ketamine were first observed in 2000 (Berman et al., 2000), an increasing number of researchers have been examining the therapeutic effects of ketamine. Ketamine has a short-term and rapid antidepressant effect. Ketamine has a high response rate (65–70%) within 24 h of use when used to treat depression (Lener et al., 2017). After 24 h, there is a significant decrease in HDRS scores in patients treated with ketamine compared to the placebo group (McGirr et al., 2015). Also, the antidepressant effects of ketamine can last for about a week (McGirr et al., 2015; Lener et al., 2017). Another interesting experiment showed a larger left NAc volume in patients with MDD compared to that in controls, but no significant enlargement in the right NAc; ketamine treatment was found to reduce the volume of the left NAc but increased the left hippocampal volume in patients achieving remission (Abdallah et al., 2017). Besides, clinical studies have revealed that ketamine also has a good anti-cocaine addiction effect. In the experiment by Dakwar et al. (2014, 2017), cocaine intake and frequency in real life are significantly reduced after ketamine injection. Therefore, ketamine may be helpful in the treatment of comorbid addiction and depression in the future. Future research needs to focus on the therapeutic effect of ketamine in comorbid depression and addiction. Moreover, other glutamate receptor antagonists should be used in future clinical studies.

In conclusion, the glutamatergic system and synaptic plasticity of glutamate neurons may play a crucial role in the generation and treatment of comorbid depression and addiction. However, more direct evidence is needed to clarify this complex relationship. Further studies on glutamate and its receptors may shed light on the mechanism underlying comorbid depression and addiction. CP-AMPAR-specific blockers as well as mGluR and NMDA receptor antagonists should be further investigated in comorbidity models to observe their effects, and more clinical studies should be conducted on these drugs.

### Transcription Factors

All kinds of drug abuse affect gene expression by changing the level of transcription factors such as CREB and ΔFosB (Sadat-Shirazi et al., 2019). Chronic drug use and withdrawal enhance the activity of CREB (Larson et al., 2015). CREB expressed in the NAc drives behavioral responses to aversive and reward stimuli, and therefore may be associated with comorbid addiction and depression (Turner et al., 2018a). Blockade of CREB activity reduces depressive-like behaviors and cocaine self-administration and seeking, but at the same time increases cocaine sensitivity in rats (Pavlovsky et al., 2013). By contrast, overexpression of CREB, which increases the excitability of NAc neurons, enhances cocaine-seeking behavior while producing depressive-like behaviors (Larson et al., 2015). Repeated cocaine administration before CSDS potentiated depressive-like behaviors in mice. This may be because chronic cocaine induces the small G protein, Ras, which in turn promotes BDNF-TrkB signaling and the subsequent activation of CREB in the NAc, thereby increasing the vulnerability to SDS (Covington et al., 2011). Furthermore, acute tramadol exposure increases the levels of MOR and phosphorylated CREB (p-CREB) in the NAc. Chronic tramadol administration in this region also results in elevated levels of MOR, ΔFosB, and p-CREB compared with saline in rats (Sadat-Shirazi et al., 2019). Increased CREB activity was found to lead to functional changes in the target gene, and CREB-mediated endogenous opioid dynorphin expression was found to be increased (Butelman et al., 2012). Dynorphin also acts on KORs, which are associated with aversive or depressive-like effects (Crowley and Kash, 2015; Carlezon and Krystal, 2016).

ΔFosB is an important transcription factor in the NAc, and a unique role has been identified for it in addiction and depression animal models. ΔFosB has been reported to promote reward-seeking and motivational behaviors, and it is closely related to drug sensitization and self-administration. Chronic administration of several antidepressant medications also induces ΔFosB in the NAc, and this induction is required for the therapeutic actions of these drugs in mouse models (Nestler, 2015). ΔFosB can influence addiction and depression by regulating the expression of specific target genes in the NAc and the brain regions that exert top-down control over NAc function, such as the PFC and HPC (Gajewski et al., 2016). The expression of specific genes has also been found to be correlated with the phenotype of animal behavior and appearance, and measuring ΔFosB has allowed us to investigate these complex relationships (Heller et al., 2014). One candidate target gene in the NAc is the opioid peptide, dynorphin, which is suppressed by ΔFosB. Enhanced ΔFosB expression through the downregulation of dynorphin may underlie the antidepressant effect, but this has yet to be confirmed experimentally (Sim-Selley et al., 2011; Nestler, 2015). Another target gene in the NAc is GluA2 (GluR2), and AMPAR subunit. ΔFosB expression can cause excessive GluR2 expression in the NAc, which decreases the responsiveness of NAc neurons to glutamate. This may explain how the induction of Δ FosB in the NAc in response to CSDS is both necessary and sufficient for resilience (Vialou et al., 2010). Since GluA2-containing AMPA channels are impermeable to Ca^2+^ and have a lower overall conductance compared to GluA2-lacking AMPA channels, chronic stress- and ΔFosB-mediated upregulation of GluA2 in the NAc could account, at least in part, for the reduced glutamatergic responses seen in these neurons of resilient mice (Nestler, 2015). This shows that increasing GluA2-lacking AMPARs in the NAc, which we see in susceptible mice, can exacerbate responses to cocaine-associated cues that promote craving and relapse in addiction models. Enhanced glutamatergic transmission in the NAc may promote vulnerability to both addiction and depression. The protein kinase CaMKII is also thought to be a key target gene. ΔFosB and CaMKII have a synergistic effect on the addiction model induced by chronic exposure to cocaine. ΔFosB binds to the CaMKII promoter and induces CaMKII expression in the NAc during cocaine treatment. CaMKII is required for the cocaine-mediated accumulation of ΔFosB in the rat NAc (Robison et al., 2013). Although ΔFosB levels are increased in the CSDS model, as well as following long-term treatment with fluoxetine, CSDS does not affect ΔFosB and CaMKII binding, while fluoxetine inhibits the binding of these two proteins through chromatin changes (Robison et al., 2014). Furthermore, viral-mediated overexpression of CaMKII in the NAc prevents the antidepressant effects of fluoxetine in the CSDS paradigm (Robison et al., 2014).

Thus, overexpression of CaMKII and ΔFosB may play a role in addiction and depression. It will be interesting to see if future studies that inhibit the expression of CaMKII and hinder its binding with ΔFosB results are successful in treating comorbid depression and addiction. Through unbiased genome-wide methods, one of the genes that has been identified to be the most robustly induced in the NAc under resilience and upon ΔFosB overexpression is *Sparcl1*, which encodes *Sparc-like 1* (also known as *hevin*, Nestler, 2015). *Sparcl1* is an anti-adhesive matrix molecule that is highly expressed in the adult rat brain. Furthermore, overexpression of *Sparcl1* in the mouse NAc exerts potent antidepressant-like effects, as measured by a decrease in the time spent immobile and an increase in the latency to immobility in the FST (Nestler, 2015). However, research on *Sparcl1* in addiction is lacking. Future studies should explore whether overexpression of *Sparcl1* plays a role in the symptoms of addiction, or whether it is overexpressed in mouse models of drug addiction.

In a clinical research study, it was demonstrated that ΔFosB is upregulated in the NAc of humans with cocaine addiction (Robison et al., 2013), and reduced in the NAc of patients with depression (Vialou et al., 2010). The same pattern of chromatin modifications and CaMKII repression is seen in the NAc of patients with depression treated chronically with antidepressant medications, but not in medication-free patients (Robison et al., 2014). Importantly, the same Robison et al. research group also demonstrated the induction of ΔFosB and CaMKII in the NAc of human cocaine addicts, suggesting possible targets for future therapeutic intervention (Robison et al., 2013). *Sparcl1* levels are downregulated in the NAc of patients with depression (Nestler, 2015), though its role in addiction is still unknown. Changes in ΔFosB can also occur in areas of the brain associated with the NAc. The study by Gajewski et al. (2016) shows ΔFosB downregulation in the HPC, the brain region that exerts the top-down control over NAc function, but not in the PFC in the brains of people with both depression and addiction. Further, they showed that potential ΔFosB transcriptional targets, including GluA2, are also downregulated in the HPC but not in the PFC of cocaine addicts (Gajewski et al., 2016). ΔFosB demonstrates different changes in the NAc and HPC in cocaine addicts, and the mechanism of these changes has yet to be elucidated. It is critical to note that the human populations included in this study lacked the homogeneity of preclinical rodents or primate models.

In conclusion, the ability of transcription factors in the NAc to control the expression of genes that are important for the onset of both addiction and depression could explain the comorbidity of these conditions. The expression of dynorphin is influenced by ΔFosB and CREB, which play an important role in activating KOR and further leading to addiction and depression. Besides, ΔFosB may also play a role in addiction or depression by affecting the expression of other target genes, GluA2, CaMKII, and *Sparcl1*. Further research is required to explore their roles in comorbid depression and addiction, and these targets could provide new treatment options in the future.

### Deep Brain Stimulation

In the present article, we have presented several common mechanisms of addiction and depression and have reported that some receptor antagonists and agonists have antidepressant and addiction-relieving effects. DBS, on the other hand, is a surgical treatment that works by placing bipolar electrodes over specific brain regions and stimulating them with an implanted pulse generator. Depending on the target brain region and the patient’s disease, the stimulus parameters are programmable (Salling and Martinez, 2016). DBS allows neural pathway activity to be regulated, which has a therapeutic effect. This method has had preliminary success, such as improved motor function in dystonia, essential tremor, and Parkinson’s disease (Salgado and Kaplitt, 2015).

Preclinical studies have supported the use of DBS in treating addictive behaviors. Studies on rodents showed that intracranial stimulation of the NAc reduces alcohol self-medication, morphine-seeking behavior, cocaine-seeking behavior, and relapse (Pierce and Vassoler, 2013). It has also been hypothesized that DBS over the NAc promotes abstinence from addictive drugs by promoting changes in the synaptic plasticity of dopaminergic neurons (Kuhn et al., 2014; Ge et al., 2018). Preclinical studies also support the use of DBS to treat depression. Accumulating evidence indicates that DBS of the NAc may decrease depressive-like behavior in the chronic unpredictable stress (CUS)-induced animal model of depression (Hamani et al., 2014; Dandekar et al., 2018). Hamani et al. found that DBS delivered to the NAc induces significant antidepressant-like effects, as assessed using the FST (Hamani et al., 2014). While these DBS studies only focused on single disease models, the DBS studies on comorbid addiction and depression rarely do. This may be because at this stage, stable animal models, which provide a good simulation of human comorbid addiction and depression, are lacking; standards to measure comorbidity in animal models are also lacking. We hope that in the future, we will be able to compare treatment with DBS in a comorbidity model with that in individual addiction or depression models to comprehensively evaluate the treatment effect of DBS and to better direct clinical research.

Compared with preclinical research, the number of clinical studies is still very limited. We summarized the clinical research findings in recent years regarding the use of DBS of the NAc to treat addiction and depression (Table 2; Schlaepfer et al., 2008; Heinze et al., 2009; Kuhn et al., 2009, 2011, 2014; Müller et al., 2009; Bewernick et al., 2010, 2012; Grubert et al., 2011; Zhou et al., 2011; Valencia-Alfonso et al., 2012; Voges et al., 2013; Millet et al., 2014; Gonçalves-Ferreira et al., 2016; Ge et al., 2018; Chen et al., 2019; Qu et al., 2019). Clinical ratings [HDRS (1960) and Montgomery and Asberg (1979) depression rating scales; Hamilton, 1960; Montgomery and Asberg, 1979] improved in all three patients when the DBS stimulator was on, and worsened in all three patients when the stimulator was turned off, in the experiment by Schlaepfer et al. (2008). Another study showed that 12 months following the initiation of DBS treatment, five patients achieved a 50% reduction in their HDRS score (Bewernick et al., 2010). The depression rating scores were significantly reduced in the study population as a whole from the first month of NAc-DBS in another study (Bewernick et al., 2012). Positron emission tomography (PET) imaging performed by Schlaepfer et al. (2008) after NAc stimulation showed a bilateral increase in metabolic activity in the dorsolateral PFC, a structure that is usually hypoactive in depression. Additionally, decreased activity was observed throughout the study in the ventromedial PFC, which has been reported to be hyperactive in depression (Drobisz and Damborska, 2019). NAc-DBS not only has the effect of treating depression but also proves effective in treating addiction. As mentioned, there is often comorbid depression among people with addiction; thus, NAc-DBS could be used to treat this comorbidity. From these reports on NAc-DBS, almost all patients who received NAc-DBS had a positive reduction in drug intake and remained abstinent for even several years (Table 2, Heinze et al., 2009; Kuhn et al., 2009, 2011; Müller et al., 2009; Zhou et al., 2011; Valencia-Alfonso et al., 2012; Voges et al., 2013; Gonçalves-Ferreira et al., 2016; Chen et al., 2019; Qu et al., 2019). Furthermore, we also found that some patients with como rbid addiction and depression developed a better mood and became more energetic during the DBS treatment process (Zhou et al., 2011; Ge et al., 2018; Chen et al., 2019). Another study focused on the features of power spectra for local field potentials (LFPs) of the NAc and the anterior limb of the internal capsule (ALIC) in heroin addicts. LFP theta power in the ALIC and alpha power in the NAc correlated with drug craving and depressive symptoms. This may illustrate the neurophysiologic characteristics of heroin addiction and its comorbidity, providing a potential theoretical basis for optimizing DBS therapy (Ge et al., 2018). Following stimulation of the NAc and ALIC simultaneously, five patients were abstinent for more than 3 years, two relapsed after abstaining for 6 months, and one was lost to follow-up at 3 months. Simultaneous DBS of the NAc and ALIC also decreased the mean pre-implantation HDRS score in the abstinent subjects, indicating improved mood after DBS. Although the HDRS scores in the two relapsed patients also decreased at the six-month follow-up, it increased to nearly the preoperative level after they relapsed. It is worth noting that the two patients who relapsed had much higher HDRS scores than the abstinent patients at each time point. This suggests that patients with comorbid addiction and depression may have a higher risk than patients with a normal mood to relapse after DBS (Chen et al., 2019). This also suggests that simultaneous DBS of the NAc and ALIC are indeed a good treatment option for addiction, but there is room for improvement in the treatment of comorbid depression and addiction. The results discussed above may be due to the limited sample size. Thus, a double-blinded study on patients with refractory opioid dependence (ROD), with larger sample size and also using HDRS to assess changes in the severity of depression, is currently in progress (Qu et al., 2019). It would be meaningful to evaluate the effect of DBS on comorbid depression and addiction by classifying and analyzing the data of subjects with this comorbidity.

**Table 1:** The relationship, some possible mechanism, specie and drug related to the comorbidity of addiction and depression.

Relationship	Possible mechanism	Species	Drug	References
A/D	VTA Cav1.3 channel mediated cocaine-related and depressive-like behavior through a NAc shell CP-AMPAR mechanism *via* GluA1 phosphorylation at S831	C57BL/6 mice	Cocaine	Martinez-Rivera et al. (2017)
D→A	Dopaminergic dysfunction in bulbectomized rats	OBX rats	CB1 receptor agonist WIN	Amchova et al. (2014)
A/D	Dopaminergic transmission in the NAc *via* D1-like receptors	Rats	Morphine	Gao et al. (2012)
A→D	DAT ↓	Heroin-dependent subjects	Heroin	Liu et al. (2013)
A/D	CHT heterozygosity → blunted DA elevations following systemic nicotine or cocaine administration.	CHT^+/–^ mice, C57BL/6J mice	Cocaine nicotine	Dong et al. (2013)
A	The expression profile of the HCN2 subunit in both glycosylated and non-glycosylated protein isoforms ↑	Sprague–Dawley rats	Cocaine	Santos-Vera et al. (2013)
D	Expression and function of the HCN2 in ChIs of NAc shell ↓	p11 conditional knockout (cKO) mice and SDS mice	/	Cheng et al. (2019)
A	Cocaine→p11 expression in the NAc↓, while p11 expression↑→cocaine conditioned place ↓	p11 knockout mice	Cocaine	Arango-Lievano et al. (2014)
D	The expression of p11↓→ depression	p11 knockout mice	/	Alexander et al. (2010) and Warner-Schmidt et al. (2012)
A/D	Silencing of GSK3β in the NAc shell →excitability of TANs↓	GSK3β knockdown rats	Cocaine	Crofton et al. (2017)
A	mGluR5-mediated reduction in GluA2-containing AMPARs at NAc shell synapses	Adult male C57BL/6J mice	Cocaine	Benneyworth et al. (2019)
A	mGluR5 antagonists → elevation of extracellular glutamate in the NAc↑ → therapeutic anti-cocaine effects	Rats	Cocaine	Li et al. (2018)
D	mGluR5-mediated signaling in the NAc ↓	mGluR5^#x02212;/–^ mice	/	Shin et al. (2015)
A/D	Blockade of the mGlu2/3 receptor in the NAc → the antidepressant-like effects	Sprague–Dawley rats	Methamphetamine	Iijima et al. (2013)
A	mGluR2/3 ↓→ neural plasticity	Sprague–Dawley rats	Morphine	Qian et al. (2019)
A	AMN082 → mGluR7(+) → the development and expression of cocaine and morphine sensitization, and the reciprocal cross-sensitization↓	Male Swiss mice	Cocaine, Morphine	Jenda et al. (2015)
D	AMN082 → mGluR7(+) → adenylate cyclase/protein kinase A activation ↓→the entry of Ca^2+^ through voltage-dependent Ca^2+^ channels ↓→ glutamate release ↓	Male Sprague–Dawley rats	/	Wang et al. (2018)
D → A	Activate the KOR→ inhibition of phasic dopamine signaling	Sprague–Dawley rats	The KOR agonist salvinorinA (salvA)	Ebner et al. (2010)
D → A	Immediate dysphoric effect of the KOR agonist salvA coincides with sensitivity to cocaine reward net ↑. Delayed effect of salvA → basal hedonic state rebound ↑ coincides with sensitivity to cocaine reward net ↓	Sprague–Dawley rats	Cocaine	Chartoff et al. (2016)
A/D	Knockdown of Pdyn within the NAc → depression-like behavior and cocaine sensitization↓	Wistar rats	Cocaine	Cohen et al. (2014)
A → D	Morphine withdrawal → Prodynorphin mRNA and protein levels ↑ → depressive-like behaviors	Male C57BL/6J mice	Morphine	Zan et al. (2015)
A/D	CREB activity ↑→ depression-like signs. Disruption of CREB activity → anti-depressant like effects and more sensitive to the rewarding effects of cocaine	Mice	Cocaine	Dinieri et al. (2009)
D	ΔFosB induction in NAc is both a necessary and sufficient mechanism of resiliency and of antidepressant responses	Human/Mice	/	Vialou et al. (2010)
A	Δ FosB is both necessary and sufficient for cocaine induction of CaMKIIα gene expression. Overexpression of Δ FosB in NAc increased behavioral responsiveness to cocaine is CaMKII dependent	Human/Rats	Cocaine	Robison et al. (2013)

*A/D indicates that it has a important role in both addiction (A) and depression (D), A → D indicates that addiction (A) induce depression (D), D → A indicates that depression (D) induce addiction (A)*.

**Table 2:** Summary of clinical deep brain stimulation (DBS) studies for drug addiction and depression.

Disorder	N	Follow-up (month)	Design	Electrode type	Amplitude (V)	Frequency (Hz)	Pulse width (μs)	Drug	Outcome	References
D	3	0.5–2	Double-blind manner	Bilateral	4	145	90	–	Clinical ratings improved, response rate = 33%.	Schlaepfer et al. (2008)
D	10	12	Open-label study	Bilateral	1.5–10.0	100–150	60–210	–	Anti-depressant and antianhedonic effects in TRD patients, response rate = 50%, remission rate = 30%	Bewernick et al. (2010)
D	10	12	Open-label study	Bilateral	1.5–10.0	100–150	60–210	–	Support cognitive safety of NAc-DBS and improve cognitive performance in patients with TRD.	Grubert et al. (2011)
D	11	12–48	Open-label study	Bilateral	5.0–8.0	130	90	–	A stable antidepressant and anxiolytic effect and an amelioration of quality of life in TRD patients, response rate = 45%, remission rate = 9%.	Bewernick et al. (2012)
D	4	15	Open-label study	Bilateral	4.0–8.0	130	60	–	NAc is a more promising target than the caudate, response rate=75%, remission rate=25%.	Millet et al. (2014)
A/D	1	12	Open-label study	Bilateral	3	130	90	Alcohol	No change in anxiety/depression, resolution of preop alcohol dependency.	Kuhn et al., 2009
A	3	12–18	Open-label study	Bilateral	3.5–4.5	130	90	Alcohol	Two remained abstinent, while one showed a remarkable reduction of days while drinking	Müller et al. (2009)
A	4	14	Open-label study	Bilateral	3.5–4.5	130	90	Alcohol	All three patients reported a marked to nearly complete reduction of craving, NAc is indeed sensitive to alcohol-related cues.	Heinze et al. (2009)
A	1	72	Open-label study	Bilateral	2.5	145	90	Heroin	Procedure refrained from drug abusing during active stimulation for the first 2.5 years. He had remained drug free for 3.5 years even after the stimulation was removed with no relapse, the Self-Rating Depression Scale and Self-Rating Anxiety Scale decreased and returned to normal ranges on DBS stimulation.	Zhou et al. (2011)
A	1	12	Open-label study	Bilateral	5.5	130	120	Alcohol	Led to a significant reduction of drug consumption and modulated associated deficits in cognitive control.	Kuhn et al. (2011)
A	1	6	Open-label study	Bilateral	3.5	180	90	Heroin	The patient first reduced his use to the weekends and then succeeded in cessation of his heroin use; he was clean for more than 6 months with the exception of a 14-day relapse.	Valencia-Alfonso et al. (2012)
A	5	31–47	Off-label study	Bilateral	4.5	130	90	Alcohol	All patients experienced significant and ongoing improvement of craving. Two patients remained completely abstinent for more than 4 years.	Voges et al. (2013)
A	2	24	Open-label study	Bilateral	4.5/5	130/140	90/120	Heroin	Both patients were yet consuming other psychotropic substances (Patient 1: alcohol and amphetamines, Patient 2: amphetamines and benzodiazepines).	Kuhn et al. (2014)
A	1	30	6 months of double-blinded 3 months of single-blinded	Bilateral	2.5–4.5	150	150	Cocaine	Posterior NAc and the bed nucleus of the stria terminalis (BNST) DBS was useful and safe for the treatment of this case of refractory cocaine dependence (RCD), and its positive effect was maintained >2.5 years after surgery.	Gonçalves-Ferreira et al. (2016)
A/D	7	3–40	Single-blinded	NAc /ALIC	ALIC = 2.0–2.5 NAc = 2.2–3.3	ALIC = 150–240 NAc = 180–240	ALIC = 185 N = 145	Heroin	For all patients, an instant positive psychobehavioral response. Theta power in the ALIC/dorsal striatum and alpha power in the NAc may be associated with drug cravings and depressive symptoms, respectively, in heroin addicts.	Ge et al. (2018)
A	8	>24	Open-label study	NAc/ALIC	1.5–7	130–185	150–240	Heroin	With DBS, five patients were abstinent for more than 3 years, two relapsed after abstaining for 6 months, and one was lost of follow-up at 3 months. The degree of cravings for drug use after DBS was reduced if the patients remained abstinent. Simultaneous DBS of the NAc and ALIC also improved the quality of life, alleviated psychiatric symptoms.	Chen et al. (2019)
A	60	6	Double-blinded study	NAc/ALIC	ALIC = 3 NAc = 3	ALIC = 165 NAc = 145	ALIC = 210 NAc = 210	Opioid	Anticipated to be concluded by July 2020.	Qu et al. (2019)

*A, addiction; D, depression; N, Number of participants; ALIC, anterior limb of the internal capsule; TRD, treatment-resistant depression; “–” data not provide*.

In summary, DBS is a possible treatment for comorbid addiction and depression. However, the mechanisms of DBS remain unclear and more experiments are still required. Nevertheless, according to the current literature, DBS of the NAc has therapeutic effects in both addiction and depression (Benabid and Torres, 2012). Data on DBS treatment in patients with comorbid addiction and depression are still needed, though the NAc represents a key target for DBS in the treatment of this comorbidity. Also, co-stimulation of the NAc and other related brain regions may have more potential for treating this comorbidity than stimulation of the NAc alone.

## Conclusions

There is a growing awareness that addiction is often comorbid with depression. Multiple epidemiological studies have found that people with addiction are at an increased risk of depression, and depressed people are at greater risk of drug addiction than non-depressed people. In this review, we found that the NAc is located at an important position in the neural circuit and is closely related to several important brain regions, such as the VTA, PFC, HPC, and LH. The neural pathways associated with the NAc are important for the regulation of addiction and depression phenotypes. The release of various neurotransmitters and the expression of proteins showed similar changes in the NAc in depression and addiction, such as dynorphin and DA. In the addicted and depressed states, the relevant transcription factors, such as CREB and ΔFosB, in the NAc are activated; the expression of the related genes, such as dynorphin, GluA2, CaMKII, and *Sparcl1*, regulated by these transcription factors also change. Moreover, DBS on the NAc has a therapeutic effect in animal models of addiction and depression separately, as well as in human clinical studies. Recent advances have found that the mechanisms underlying comorbid depression and addiction at anatomical, physiological, cellular, and molecular levels of the brain are closely linked to changes in the NAc (Table 1), a key brain region in the regulation of rewards and motivational behavior. Therefore, we believe that the NAc is a key site for the study of comorbid mechanisms and the treatment of comorbidity.

Although addiction and depression have overlapping neural circuits and similar molecular mechanisms, the exact mechanism underlying this comorbidity is still unclear because the current studies still have many limitations. First, there is a lack of a mature comorbidity model (Table 3). From the literature we reviewed, we found that there are plenty of studies on models of addiction and depression separately. However, when it comes to the comorbidity model of addiction and depression, there are limited studies. Also, the comorbidity model is dominated by male animals, and there are relatively few experiments on female animals as research subjects. Second, some changes in neurotransmitters and proteins as well as the therapeutic effect of some drugs have yet to be measured in existing comorbidity models, even though they have been found to have similar changes or therapeutic effects in depression and addiction models, separately. This makes it hard to determine their exact role in comorbidity and find differences between comorbidity and single disease. Third, although sporadic clinical studies have measured changes in certain proteins like p11 and GSK3β in patients with depression or addiction, the results are similar to those in animal studies. However, there is still a lack of critical and sufficient evidence for the role of these proteins in human comorbidity. Fourth, some clinical studies lack a comprehensive evaluation of patients, which may exclude some cases with comorbidity.

**Table 3:** The types of animal model in the comorbidity of depression and addiction.

Disorder	Model	Characteristic	Reference
Addiction	Self-administration (SA)	The animals were trained to obtain an intravenous fluid injection by performing operant response, for example pressing a lever or the inserting its snout into a hole.	Li et al. (2018)
Addiction	Intracranial self-stimulation (ICSS)	This procedure is based on the observation that rats will press a lever to pass a small current through electrodes located in various brain areas.	Melis et al. (2005)
Addiction	conditioned place preference (CPP)	A Pavlovian conditioning procedure inwhich the animal learns to prefer an environment that is paired with drug effects.	Siahposht-Khachaki et al. (2016)
Depression	Chronic social defeat stress (CSDS) model	CSDS simulates the pathogenesis of depression at a social level. With a frequently performed stimulation, model rodents manifest a stress response to produce long-term behavioral and psychosocial change.	Koo et al. (2019)
Depression	Olfactory bulbectomy (OBX) model	The bilateral ablation of the olfactory bulbs was performed, it is a well-established model of depression with high face, construct, and predictive validity which closely mimics neurochemical, neuroanatomical, behavioral and endocrine changes seen in patients with major depression.	Babinska et al. (2016)
Depression	p11 KO model	p11 loss within neurons of the NAc induces depression-like behaviors.	Alexander et al. (2010)
Depression	The KOR agonies induce depression-like behavior	SalvA increased ICSS thresholds and significantly lowered breakpoint on the progressive ratio schedule, indicating a decrease in motivation.	Ebner et al. (2010)
Depression	Chronic restraint stress	Animals are placed in restraint tubes for several hours daily, repeated over several days.	Thompson et al. (2015)
Comorbidity	The KOR agonist +CPP	Stress-induced activation of KOR by endogenous dynorphin opioids may enhance the rewarding valence of drugs of abuse by potentiating the evoked dopamine response.	Ehrich et al. (2014)
Comorbidity	Chronic mild restraint (CMR)+morphine-CPP	Rats undergoing CMR, which were evaluated for novelty-seeking, forced open-space swimming, and locomotor activity to validate CMR as a depression- like model Rats undergoing CMR were trained for morphine-induced CPP.	Gao et al. (2012)
Comorbidity	OBX model + intravenous self-administration	A rat model of depression and addiction dual disorder where olfactory bulbectomized animals was developed and it showed a significantly higher vulnerability in methamphetamine intravenous self-administration (IVSA) paradigm.	Babinska et al. (2016)
Comorbidity	CSDS + SA	The activational effect of ostensibly aversive events such as social stress on the acquisition of cocaine self-administration, an intensely reinforcing event, prompts a closer scrutiny of the behavioral features that may inform on the reinforcing efficacy of the drug.	Miczek and Mutschler (1996)
Comorbidity	CSDS+CPP	SDS has also been shown to enhance the sensitivity to cocaine CPP.	Ribeiro Do Couto et al. (2009)
Comorbidity	GSK3β knockdown	Knocked down GSK3β expression with a novel adeno-associated viral vector (AAV2) and assessed changes in anxiety- and depression-like behavior and cocaine self-administration in GSK3β knockdown rats.	Crofton et al. (2017)
Comorbidity	P11 KO +SA	It may be a comorbid model, but experiments are needed to prove whether it can be a comorbid model.	Warner-Schmidt et al. (2012)
Comorbidity	CHT^+/–^ mice+SA	It may be a comorbid model, but experiments are needed to prove whether it can be a comorbid model.	Dong et al. (2013)

Despite these limitations, performing some of the proposed experiments in the future would contribute to confirming that the NAc as a critical treatment site of comorbid addiction and depression. First, future studies should consider how repetitive the self-administration of drugs or other addiction models can lead to depressive-like behavior to create comorbidity models because most current comorbidity models are based on depression models. What’s more, some models like the CHT^+/–^ model should be assessed in the behavioral phenotypes of addiction and depression to further select the models that can be used to establish comorbidity models. Also, female animals should be included in more studies of comorbidity to clarify the influence of gender on comorbidity. It is imperative to look for a mature comorbidity model by finding differences and commonalities in comorbidity models that are made *via* different methods in the future. Second, future studies need to add experiments on comorbidity models to measure changes of proteins, transmitters, transcription factors, and their regulated gene expression in the NAc. It is also necessary to apply therapeutic drugs and DBS to comorbidity models and observe their therapeutic effects on addiction and depression. Third, in clinical studies, patients need to be comprehensively evaluated. It is better to divide patients into patients with addiction but no depressive symptoms, patients with depression but no drug addiction, and patients with comorbidity. Then, based on preclinical studies, changes in key proteins, neurotransmitters, transcription factors, and the therapeutic effect of drugs and DBS should be further measured in the NAc of these three types of patients to supplement the clinical trial data.

If we can understand the complex pathogenesis between addiction and depression in the NAc, this could be used to stop the vicious cycle of these two diseases aggravating each other. This could effectively prevent the risk of drug addiction in patients with depression and help to treat patients with addiction by reducing the negative emotions caused by the treatment process and reducing the risk of concurrent depression.

## Author Contributions

LX wrote the first draft of the manuscript. JN and YL wrote sections of the manuscript. All authors contributed to manuscript revisions as well as read and approved the submitted version.

## Conflict of Interest

The authors declare that the research was conducted in the absence of any commercial or financial relationships that could be construed as a potential conflict of interest.
